# Frequency of CD4+CD25+Foxp3+ cells in peripheral blood in relation to urinary bladder cancer malignancy indicators before and after surgical removal

**DOI:** 10.18632/oncotarget.7199

**Published:** 2016-02-05

**Authors:** Wojciech Jóźwicki, Anna A. Brożyna, Jerzy Siekiera, Andrzej T. Slominski

**Affiliations:** ^1^ Department of Tumour Pathology and Pathomorphology, Nicolaus Copernicus University Collegium Medicum in Bydgoszcz, Bydgoszcz 85-796, Poland; ^2^ Department of Tumour Pathology and Pathomorphology, Oncology Centre-Prof. Franciszek Łukaszczyk Memorial Hospital, Bydgoszcz 85-796, Poland; ^3^ Department of Urology, Oncology Centre-Prof. Franciszek Łukaszczyk Memorial Hospital, Bydgoszcz 85-796, Poland; ^4^ Departments of Dermatology and Pathology, University of Alabama at Birmingham, Birmingham, AL 35294, USA; ^5^ VA Medical Center, Birmingham, AL 35233, USA

**Keywords:** Treg, urothelial bladder cancer, histological malignancy indicators, immune escape, tumor progression

## Abstract

Tumor cells communicate with stromal cells, including cancer-associated fibroblasts (CAFs) and tumor-associated macrophages (TAMs), to form microenvironment inhibiting immune responses. Regulatory T cells (Tregs, CD4+CD25+FoxP3+) stimulate immune tolerance and facilitate tumor progression. We analyzed the changes in Treg frequencies assessed using flow cytometry in the peripheral blood of patients with urothelial bladder cancer before and after tumor-removal. Changes in Treg frequency were investigated in relation to clinicopathomorphological indicators of tumor malignancy and expression of RCAS1 on CAFs and TAMs. Higher Treg frequencies were observed in early phase of tumor growth (pTa-pT2), in larger tumors, with more aggressive type of invasion, and with expression of RCAS1. The later phase of tumor development, accompanied by a nonclassic differentiations and pT3-pT4 advancement, had lower number of tumor infiltrating lymphocytes (TILs) and lower Treg frequency. Furthermore, in pT2-pT4 tumors, a decreased post-surgery Treg frequency was associated with poorer prognosis: patients with the lowest frequency of Tregs died first. These findings strongly suggest that the Treg frequencies at later phase of tumor growth, associated with a low anti-tumor response, represent a new and important prognostic indicator in urinary bladder cancer.

## 1 INTRODUCTION

One of the most important characteristics of tumor cells is their ability to escape the immune surveillance through inhibition of immune responses, and induction of immunological tolerance [1–3]. Furthermore, tumor cells communicate with stromal cells, including cancer-associated fibroblasts (CAFs) and tumor-associated macrophages (TAMs), to form a specific microenvironment that provides a niche that facilitates tumor growth and progression [4]. The number of CAFs and/or TAMs correlates with markers of histological aggressiveness, such as pT stage, grade, and number of nonclassic differentiations (NDN; nonclassic differentiation number) [5, 6]. In addition, the expression of the receptor-binding cancer antigen present on SiSo cells (RCAS1) in the bladder cancer associated CAFs and TAMs is associated with a poor prognosis [7, 8, 9]. This phenomenon appears to be dependent on the RCAS1 modulation of local immunological tolerance, enabling tumor cells to escape the immune surveillance and leading to tumor progression [10, 11].

Regulatory T cells (Tregs, CD4+CD25+FoxP3+) also stimulate immune tolerance and facilitate tumor progression [4, 12]. Tregs inhibit proliferation of CD8+ cytotoxic T cells and stimulate maturation of dendritic cells [13], although the exact mechanism of the action of Tregs in carcinogenesis is unknown. Increased frequency of Tregs in the peripheral blood is often observed in various tumors such as hepatocellular, breast, ovarian and lung cancers, and malignant melanomas, and is associated with poor prognosis [14–21]. It was also suggested that Tregs accumulating in tumors and in the peripheral blood of cancer patients suppress immune antitumor responses and promote tumor growth [22–27]. However, the contribution of Tregs to some tumors such as colon cancer is not fully understood, and their role in cancer biology is controversial [28]. Since a role of Tregs in the urothelial bladder cancer biology has not been investigated, we analyzed Treg frequency in peripheral blood before and after surgery in pT2–pT4 bladder tumors. The control group consisted of patients with pTa–pT1 urothelial cancer. The specific aim of our study was analyzing the blood Treg frequency in relation to pathomorphological markers of malignancy, such as grade (G), pT, TIT, NDN, metastasis, tumor size, and the likelihood of death as well as in relation to the expression of RCAS1 in tumor cells and in TAMs and CAFs. In this study, we attempted to obtain potentially significant information about fluctuations in peripheral blood Treg frequency in relation to urinary bladder cancer malignancy, before and after surgery.

## 2 RESULTS

### 2.1 Blood Tregs and Tumor-Infiltrating Lymphocytes (TILs) in urinary bladder cancer

#### 2.1.1 Tregs relative to time from surgical removal

**Figure 1:**
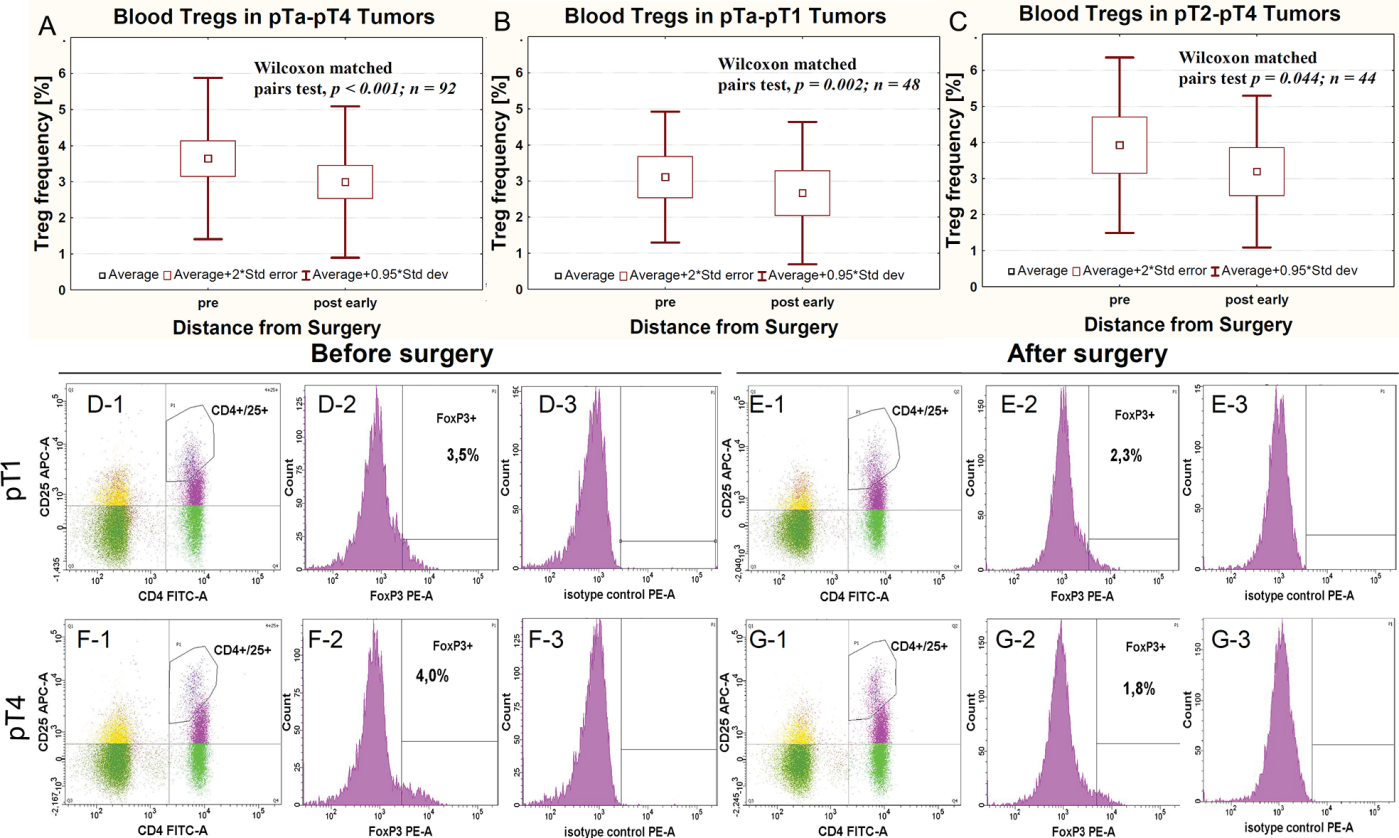
Average Treg frequency in peripheral blood relative to surgery in all pTa-pT4 cancers (**A**) and in cases subgrouped as pTa-pT1 (**B**) and pT2-pT4 (**C**). Representative Treg frequency in patient with pT1 (**D-1, D-2, D-3, E-1, E-2, E-3**) and pT4 (**F-1, F-2, F-3, G-1, G-2, G-3**) urinary bladder cancers, evaluated before (**D-1, D-2, D-3, F-1, F-2, F-3**) and after surgery (**E-1, E-2, E-3, G-1, G-2, G-3**). The **D-1, E-1, F-1** and **G-1** panels show the gate on double-positive CD4+ CD25+ cells. Blue dots represent CD4+ CD25+FoxP3+ cells. The **D-2, E-2, F-2** and **G-2** panels show the histogram representing FoxP3 staining (FoxP3+). The gates within Figures **D-1, E-1, F-1** and **G-1** represents CD4+ CD25^high^+FoxP3+ cells. Figures **D-3, E-3, F-3** and **G-3** show isotype controls used for gating FoxP3-positive cells.

The average Treg-pre frequency in pTa-pT1 and pT2-pT4 tumors showed statistically non-significant differences (Table 1). The average Treg frequency in all pTa–pT4 tumors was significantly higher before (Treg-pre) than after surgical removal of the tumor (radical cystectomy or *transurethral* resection of the tumor (TUR-Tu, Treg-post early)) (Figure 1A). The same trend was observed for both pTa–pT1 (Figure 1B) and pT2–pT4 (Figure 1C) tumors. Representative Treg frequency in patient with pT1 and pT4 tumors is presented in Figure 1D–1G.

**Table 1:** Mean Treg frequency in peripheral blood of urinary bladder cancer patients

Feature	Treg-pre [%][n / average / SD]	Treg-post early [%][n / average / SD]	Treg-post late [%][n / average / SD]
pT			
pTa-pT1	48 / 3.13 / 2.02	48 / 2.69 / 2.21	8 / 3.90 / 3.28
pT2-pT4	44 / 3.95 / 2.44	44 / 3.29 / 1.98	38 / 3.60 / 3.16
	*p* > 0.05	*p* > 0.05	*p* > 0.05
TIT*			
FR/FO	34 / 3.85 / 2.65	34 / 2.87 / 2.03	34 / 3.44 / 3.21
NE/ST/DI	10 / 4.60 / 2.56	10 / 4.60 / 2.54	8 / 4.08 / 3.08
	*p* > 0.05	*p* < 0.05	*p* > 0.05
Tumor size			
≤ 25 cm^3^	25 / 3.65 / 2.99	25 / 2.63 / 1.94	20 / 2.72 / 2.24
> 25 cm^3^ and≤ 99 cm^3^	15 / 4.49 / 2.27	15 / 4.26 / 1.75	13 / 3.94 / 2.69
> 99 cm^3^	5 / 6.40 / 6.65	5 / 3.76 / 1.79	5 / 3.71 / 1.18
	*p* > 0.05**	*p* < 0.05**	*p* > 0.05**
Metastasizing			
pN = 0	24 / 3.98 / 2.81	24 / 3.44 / 1.88	24 / 3.34 / 2.39
pN > 0	20 / 3.25 / 2.31	20 / 2.16 / 1.92	20 / 3.20 / 4.07
	*p* > 0.05	*p* < 0.05	*p* > 0.05
Survival			
Survivors	38 / 3.83 / 2.61	38 / 3.13 / 1.98	32 / 3.45 / 2.53
Not-survivors	6 / 3.05 / 2.64	6 / 1.45 / 1.26	6 / 3.61 / 5.84
	*p* > 0.05	*p* < 0.05	*p* > 0.05
NDN			
NDN = 0	18 / 3.56 / 2.75	18 / 4.40 / 1.79	12 / 3.45 / 2.22
NDN > 0	26 / 4.00 / 2.41	26 / 2.86 / 2.09	26 / 3.62 / 3.54
	*p* > 0.05	*p* < 0.05	*p* > 0.05
RCAS1 in tumor cells			
Absent	16 / 2.50 / 2.02	16 / 3.19 / 1.65	16 / 3.37 / 2.53
Present	23 / 4.55 / 2.65	23 / 3.32 / 2.24	23 / 3.76 / 3.51
	*p* < 0.05	*p* > 0.05	*p* > 0.05
RCAS1 in BPs TAMs			
Absent	15 / 2.70 / 2.37	15 / 3.23 / 1.77	15 / 2.35 / 1.95
Present	21 / 4.80 / 2.47	21 / 3.28 / 2.40	21 / 4.45 / 3.67
	*p* < 0.05	*p* > 0.05	*p* < 0.05
RCAS1 in CPs CAFs			
Absent	11 / 4.32 / 3.58	11 / 3.05 / 2.06	11 / 2.43 / 1.34
Present	14 / 4.22 / 1.78	14 / 3.94 / 2.14	14 / 5.83 / 3.83
	*p* > 0.05	*p* > 0.05	*p* < 0.05

* Refers to invasive pT2-pT4 tumors,

** Refers to comparison between tumor ≤ 25 cm^3^ and > 99 cm^3^

#### 2.1.2 Tregs and pathomorphological features

**Figure 2:**
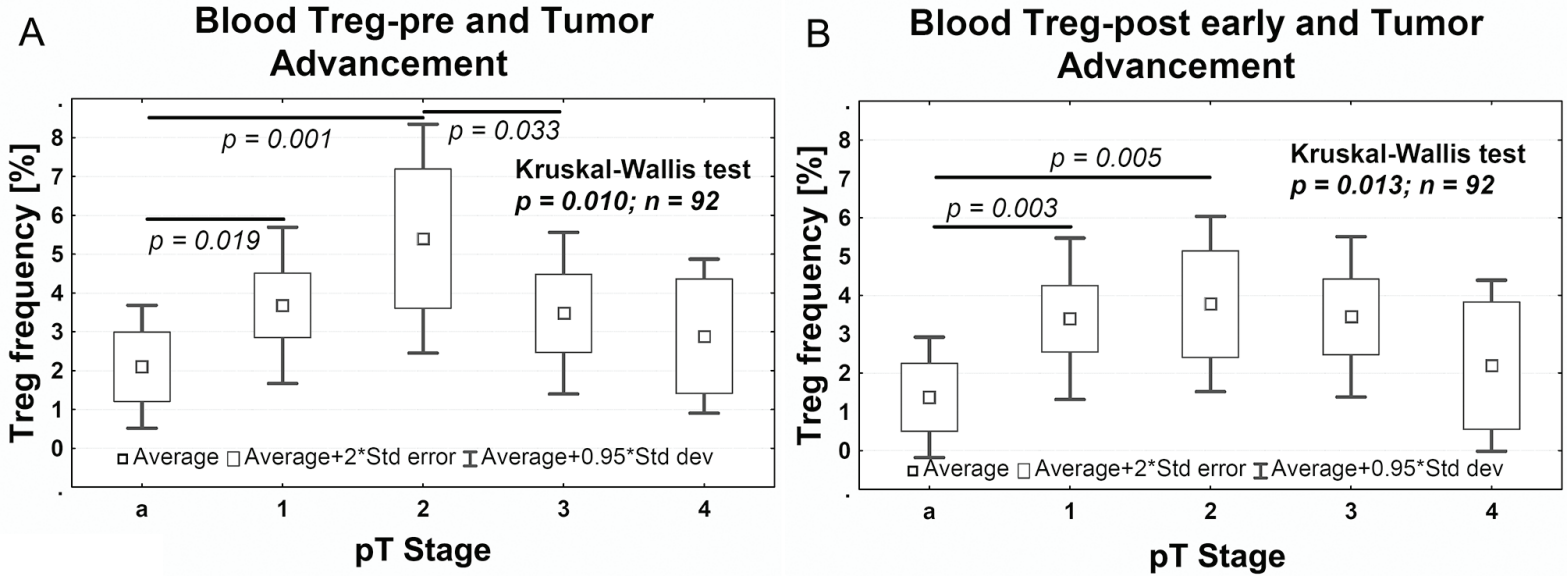
Average Treg frequency in peripheral blood relative to the pTa–pT4 progression of tumors before (**A**, Treg-pre) and after (**B**, Treg-post early) surgery. The Dunn's post hoc test followed significant Kruskal-Wallis test to compare Treg frequency between pT stages.

Treg-pre frequency significantly increased in pT1 tumors vs pTa tumors (Dunn's test, *P* = 0.003), and reached the highest frequency in pT2 tumors (Dunn's test, *P* = 0.005). In contrast, in pT3 tumors, the Treg-pre frequency was significantly lower in comparison to pT2 tumors (Dunn's test, *P* = 0.033) (Figure 2A). A similar relationship between Tregs and the pT progression was also observed after the surgery, with Treg-post early frequency being higher in pT1 and pT2 than in pTa tumors (Figure 2B).

**Figure 3:**
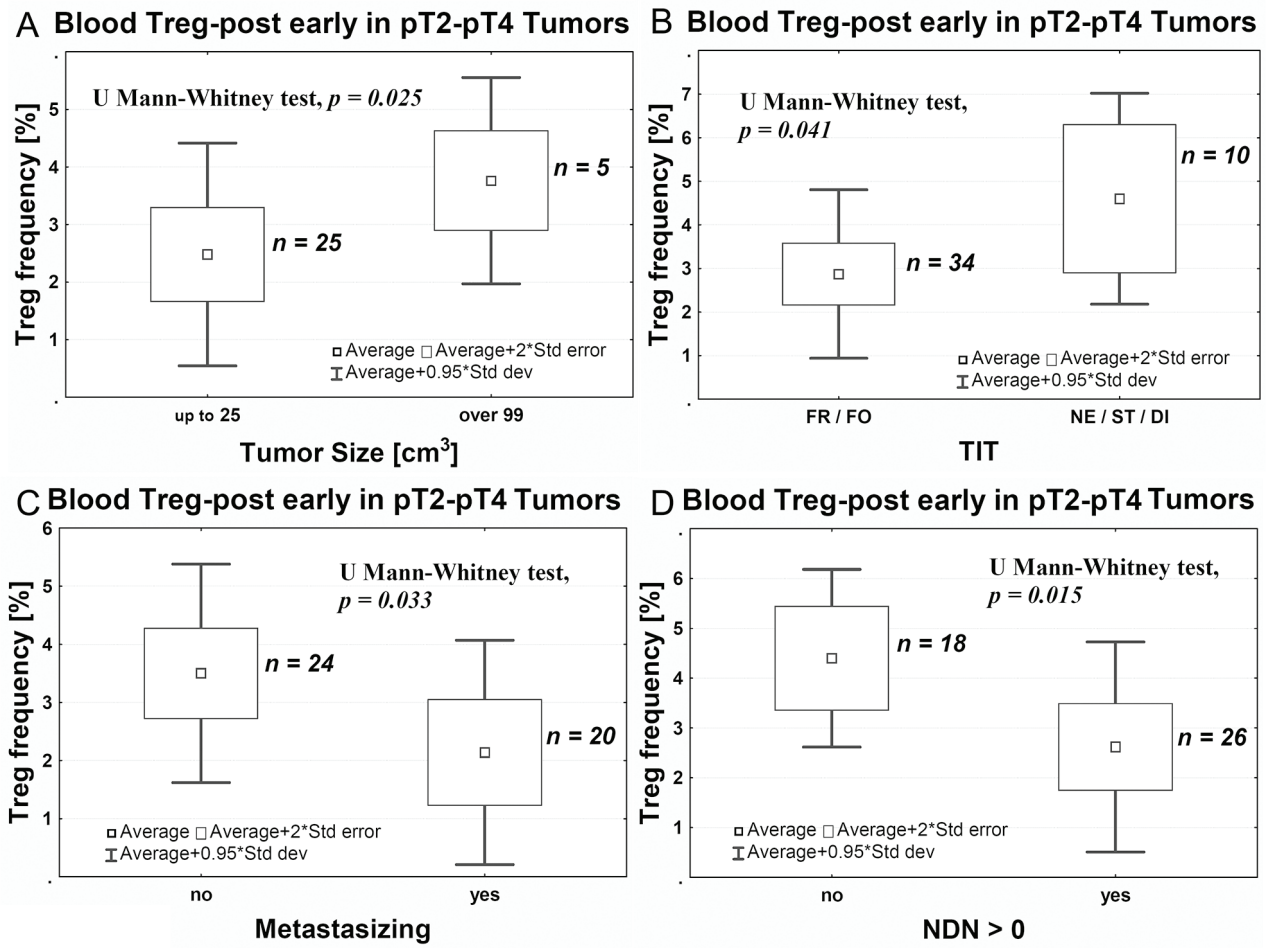
Average Treg-post early frequency in peripheral blood relative to tumor size (**A**), the aggressiveness of tissue invasion (TIT, **B**), the ability to metastasize (**C**), and ability of nonclassic differentiation (**D**). TIT, tissue invasive type; FR, frontal; FO, focal; Ne, nested; ST, styloid; DI, dispersive; NDN-non classic differential number.

Bigger size of pT2–pT4 tumors was associated with higher frequency of Tregs after surgery (Figure 3A), while small size of the tumor was associated with a sustained depletion of Tregs after surgery within the range of Treg-post early (evaluated 1 day after the surgical treatment) and Treg-post late (evaluated 7–10 days after the surgical treatment) values compared with Treg-pre (*p* = 0.029, data not shown). Furthermore, muscle invasive tumors (pT2-pT4) demonstrating histologically more aggressive type of invasion (nested, styloid and/or dispersive) were associated with significantly higher Treg-post early frequency (Figure 3B). After surgery, the average Treg-post early frequency was higher in nonmetastatic vs metastatic tumors (Figure 3C) and in classically differentiated tumors than they were in tumors with one or more nonclassical differentiates (NDN > 0) (Figure 3D). There was no significant correlation between Treg frequency and tumor grading (not shown). Analysis of the Treg frequency before and after tumor-removal surgery showed a significant decrease in the Treg frequency after TUR-Tu or radical cystectomy in both pTa (U Mann-Whitney test, *p* = 0.004) and pT2 (U Mann-Whitney test, *p* = 0.030) tumors, respectively (data not shown).

#### 2.1.3 TILs and staging

**Figure 4:**
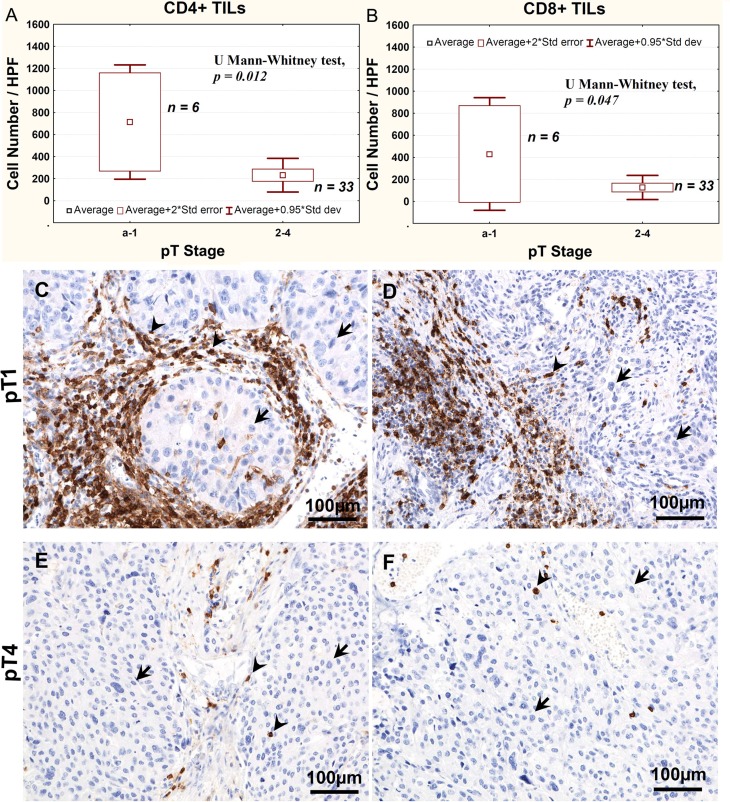
Average CD4+ (**A**) and CD8+ (**B**) TILs number, as determined using immunohistochemistry, relative to the stage of bladder cancer progression. Representative immunostaining of CD4 (**C**, **E**) and CD8 (**D**, **F**) lymphocytes in pT1 (**C**, **D**) and pT4 (**E**, **F**) tumors. Arrows point at nuclei of tumor cells, arrow heads point at CD4-positive or CD8-positive cells.

Analysis of tumor-infiltrating lymphocytes using immunohistochemistry in tissue sections revealed significantly higher number of both CD4+ and CD8+ lymphocytes in pTa-pT1 tumors than in more advanced cancers (Figure 4A–4F).

### 2.2 Treg frequency and survival time

**Figure 5:**
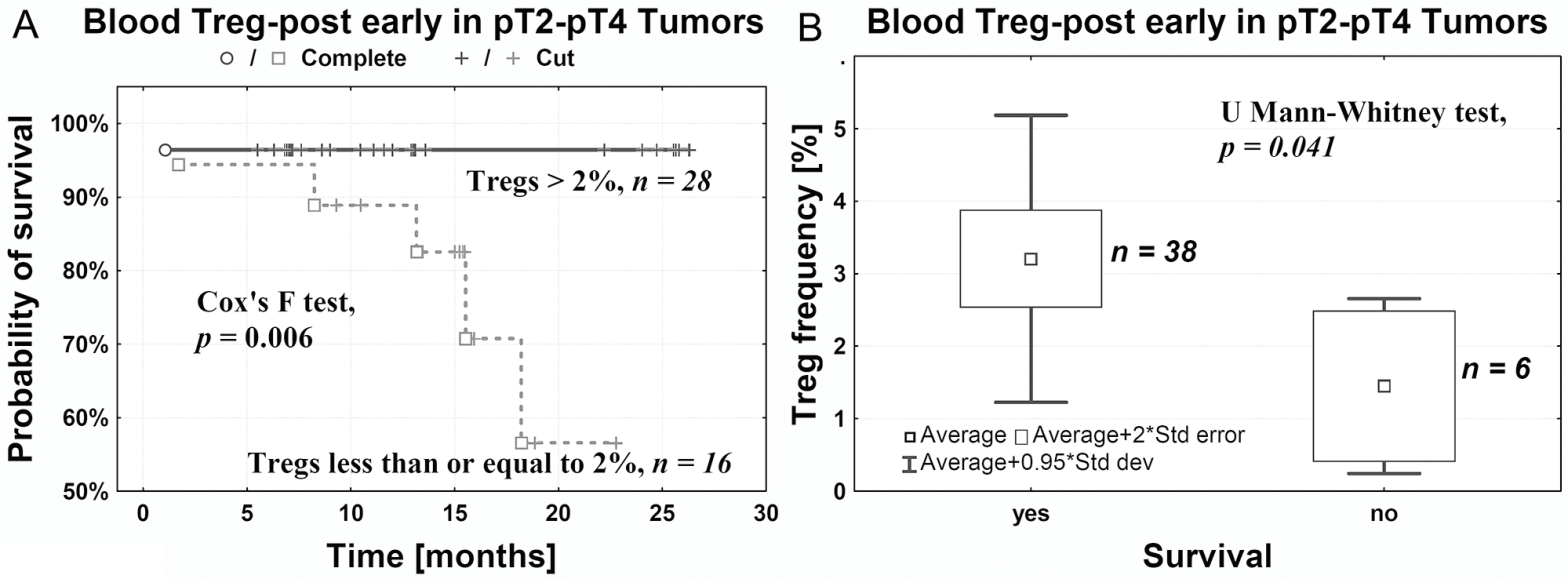
Probability of survival after cystectomy is positively correlated with Treg-post early frequency in peripheral blood (**A**) and negatively with death rate (**B**).

In patients who underwent cystectomy and whose Treg-post early frequency did not exceed 2%, we observed a significantly lower probability of survival compared with patients having higher Treg-post early frequency (Figure 5A). As relates to pT2–pT4 tumors, the death rate was significantly correlated with a decrease of Treg-post early frequency < 2% (Figure 5B).

### 2.3 Treg frequency and expression of RCAS1

**Figure 6:**
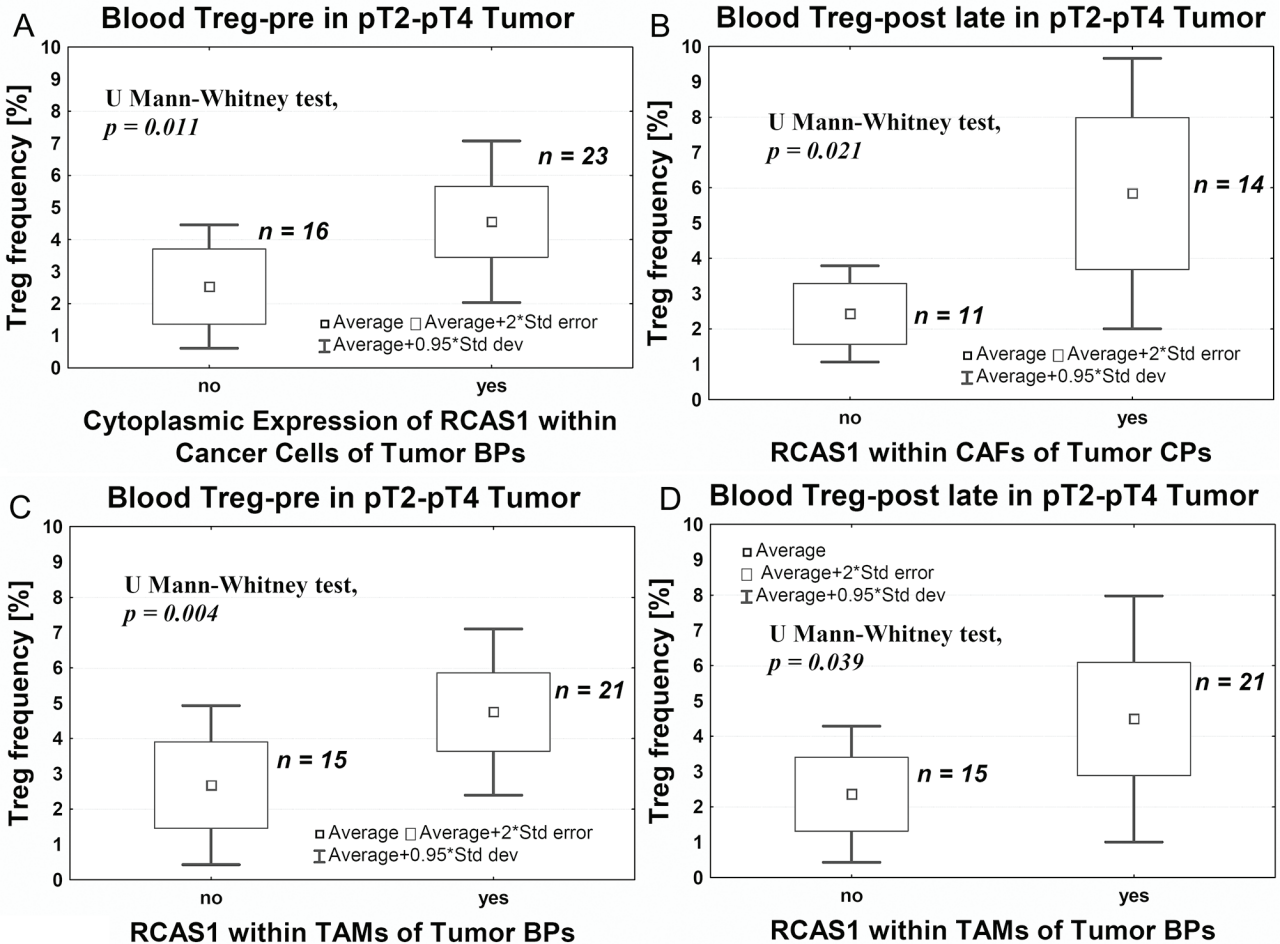
Average Treg-pre frequency in peripheral blood relative to RCAS1 expression assessed using immunohistochemistry within cancer cells of tumor BPs (**A**), CAFs in tumor CPs (**B**), TAMs in tumor BPs: before (**C**) and long after (**D**) surgery.

In pT2–pT4 tumors, we observed an increase in Tregs frequency before surgery (Treg-pre) in tumors with cytoplasmic expression of RCAS1 in the border parts (BPs) of the tumor, characterized by a high mitotic index, the presence of small nests of tumor cells, inflammatory infiltration, and/or type of stromal modeling pattern (Figure 6A). Correspondingly, in the central parts of the tumors (CPs) the presence of CAFs expressing RCAS1 within the tumor stroma was significantly associated with higher frequency of Tregs over a longer period after surgery (Treg-post late) (Figure 6B). Similarly, higher frequencies of Tregs were both observed prior to surgery (Treg-pre) and after longer postsurgery period (Treg-post late), and they were significantly associated with the presence of tumor-associated macrophages in the border parts of the tumor (BP-TAMs) expressing RCAS1 within the tumor stroma (Figure 6C, 6D). The definition of CPs is presented in Materials and Methods section. Summary of results is presented in Table 1.

## 3 DISCUSSION

In our study, we observed higher Treg-pre frequency compared with Treg-post early frequency in peripheral blood of all patients with urothelial bladder cancer, regardless of its progression (Figure 1A), which is consistent with the findings on other tumors, such as esophageal, gastrointestinal, pancreatic and breast cancers, and myeloid leukemia, as described in the literature [reviewed in [27], 15, 29]. Despite the biological differences between tumors that infiltrate the muscle vs those that do not [30, 31], an increase in the percentage of blood Tregs was observed both in pTa–pT1 tumors (Figure 1B) and pT2–pT4 tumors (Figure 1C). Naturally occurring CD4+CD25+ T cells, which are derived from the thymus, represent a small percentage (up to 5%–6%) of all CD4+ T cells, and are primarily out of antigen-specific stimulation [32, 33]. Their activation for suppression of antitumor response, needs stimulation by tumor antigen which causes their transformation [34–37] into antigen-specific Tregs, which can then induce local tumor-specific immune tolerance [33, 38–41]. These Tregs represent 1–4% of peripheral blood Tregs [15, 42]. The peripheral blood constitutes the most important source of Tregs and the percentage of Tregs probably depends directly on the presence of a tumor, because Treg frequency correlate with the number and activity of CD4+ effector T cells involved in the antitumor response (activation via the TCR receptor) [43] and the stimulating activity of tumor antigens [34–37]. This is the most likely explanation for the observation that, after surgical tumor removal, frequency of Tregs decreases, as it has been observed in ovarian cancers [15]. In our study, the Treg-pre frequency was slightly higher in tumors invading muscle (pT2–pT4) than in noninvasive papillary (pTa) or non-muscle invasive (pT1) tumors. This could be associated with evolution of immune responses involving initially stronger antitumor response followed by increased activity of Tregs, which suppress this response. The tumor-specific immune tolerance that develops impairs the immune response to the tumor [13, 41, 44] and prevents the supervision of the neoplastic process by the immune system [13]. This promotes the growth and progression of tumors [45–47]. The increased activity of Treg-pre during progression from pTa to pT2 may reflect such promotion (Figure 2A). This has directly been associated with the two succeeding stages of progression of urothelial bladder cancer, i.e., the first stage (during which a noninvasive papillary lesion (pTa) progresses to invasion) and the second stage (during which cancer cells invade the muscle layer in the bladder wall (pT2)). Increases in Treg-pre frequency have complemented previously described changes in expression of the OCT4A phenotype of tumor cells [48] as indicators of malignant progression in urothelial bladder cancer.

The analysis of Treg frequency on day 1 after surgery (Treg-post early), despite the resection of the tumor, shows significant variations between the pTa, pT1, and pT2 tumors (Figure 2B). It is possible that the observed increases in Treg-post early frequency are related to the short-term postsurgical tumor immunity [49], which is proportional to the advancement of the tumor that has been removed, but is not observed during the analysis of Treg-post late frequency. This mechanism, apparently involving large tumors (Figure 3A), requires further studies. The Treg-post early frequencies are also higher in muscle invasive tumors showing more aggressive type of invasion (Figure 3B). The observed effect may be associated with a stronger immunogenicity of the tumors, due to a better presentation of tumor antigens to the immune system during a more dynamic invasion and as a consequence of greater destruction of tumor stroma. Based on the above, we propose that the blood Tregs inhibit antitumor responses in the early phase (pTa to pT2) of urothelial bladder cancer development and in more advanced tumors with more aggressive type of invasion (TIT).

The analysis of Treg-post early frequency in relation to NDN also yields interesting results. Urothelial tumors with initially classical differentiation [30], such as invasive tumors, particularly pT2–pT4, exhibit features of numerous nonclassic differentiation patterns [50]. The decreases in number of CD4+ and CD8+ tumor-infiltrating lymphocytes (TILs) develop along the tumor advancement (Figure 4), indicating lower anti-tumor response. The lower Treg-post early frequency in NDN > 0 tumors (Figure 3D), indicating a greater risk of metastatic disease [51–53] and death [5, 50, 51], may be associated with a lower immunogenicity acquired by these tumors during the formation of nonclassic differentiation markers. It is also likely that similar mechanism underlies a significant decrease in Treg-pre frequency in pT3–pT4 tumors (Figure 2A), since 81% of them exhibit nonclassic differentiation [53]. Our observation of significantly lower Treg-post early frequency in metastatic tumors (Figure 3C) suggests that at later phases of tumor progression (defined by increased invasiveness, metastases and nonclassic differentiations) the tumor immunogenicity would decrease. Thus, a proper interpretation of the Treg frequency in peripheral blood would require its association with the phase of tumor growth.

We noted that a decrease in Treg-post early below 2% was associated with a lower probability of survival compared with patients whose Treg frequency after surgery was higher than 2% (Figure 5). This substantial decrease in Treg-post early frequency may indicate development of tumor-specific immune tolerance during tumor progression and advancement (Figures 2B and 4). Thus, the Treg frequency after surgery may be of prognostic value in urothelial bladder cancer. However, additional studies are needed on a larger group of patients to confirm the above interpretation.

We also observed a significantly higher Treg-pre frequency in pT2–pT4 tumors that expressed RCAS1 in cells located in its peripheral parts (BPs) (Figure 6A). In our previous study, we demonstrated a link between the expression of RCAS1 and the process of urothelial tumor escape from immune surveillance [6]. Higher Treg-pre frequency and an expression of RCAS1 in tumor cells might be part of the same mechanism leading to tumor evasion immune surveillance. Also the presence of RCAS1-positive macrophages in the peripheral parts of the tumor was significantly associated with higher Treg frequency, both before (Treg-pre) (Figure 6C) and long after (Treg-post late) surgery (Figure 6D). These observations suggest that RCAS1-positive macrophages promote an immunosuppressive microenvironment [54] by activating Tregs, both when the primary tumor is present (Treg-pre) and long after its excision (Treg-post late). A similar observation was made in relation to CAFs. High Treg-post late frequency coexisted with the expression of RCAS1 within the fibroblasts of the central area of the tumor (CP) (Figure 6B). It is possible that TAMs and CAFs are the most important promoters of Tregs polarization over a longer period after tumor excision.

The frequency of peripheral blood Tregs correlated with defined phases of bladder cancer progression with higher Tregs frequency accompanying early, while lower Tregs accompanying later phases of tumor growth, respectively. Lower Treg frequency at later phase of tumor growth, associated with a low anti-tumor response, represents a new and important prognostic factor in urinary bladder cancer, because patients with the lowest post-early frequency of Tregs died first. These associations encourage further studies on the mechanism(s) underlying low anti-tumor response at the later phase of urothelial bladder cancer growth with possible involvement of nonclassic differentiations and TAMs and CAFs activities.

## 4 MATERIALS AND METHODS

### 4.1 Patients and pathomorphological assessment

This study included 46 patients with urothelial bladder cancer who underwent cystectomy (or cystoprostatectomy), and 46 patients with urothelial bladder cancer who underwent transurethral resection of the tumor (TUR-Tu). Intravesical chemotherapy with Mitomycin C using a system of EMDA (electromotive drug administration) were used in 8 patients (men) just before TUR-Tu (2 and 6 patients with pTa and pT1 tumors, respectively) and in 1 patient (with pT4 tumor) just before cystoprostatectomy. The pathomorphological characteristics of the patients included in this study are presented in Table 2. Patient's survival was monitored at least for 1 year after surgery. The study was approved by the Committee of Ethics of Scientific Research of Collegium Medicum, Nicolaus Copernicus University, Poland. Tumors were diagnosed and classified according to the International Union Against Cancer TNM Classification criteria [30, 55, 56]. Briefly, pTa tumors were confined to urothelium, pT1 tumors were confined to mucosa, pT2 tumors invaded muscularis propria, pT3 tumors invaded perivesical fat and pT4 tumors - perivesical organs [55, 56]. Patients with N0–Nx tumors qualified for radical cystectomy, and patients with metastases to regional lymph nodes (N+) without clinically detectable metastases (M0) and comorbid serious internal diseases qualified for radical cystectomy followed by adjuvant chemotherapy, according to the Guidelines on Muscle-invasive and Metastatic Bladder Cancer of the European Association of Urology [57].

**Table 2:** Clinicopathomorphological characteristics of patients with urothelial bladder tumor

Feature	Number
**pTa-pT1 cohort**	**48**
Age (SD)	64 yrs (9 yrs)
Gender	
F	16
M	32
pT	
pTa	18
pT1	30
Grade	
Low grade	36
High grade	12
NDN	
NDN 0	44
NDN 1	3
NDN 2	1
NDN ≥ 3	0
TIT*	
FR/FO	26
NE/ST/DI	4
**pT2-pT4 cohort**	**44**
Age (SD)	63 yrs (7 yrs)
Gender	
F	4
M	40
pT	
pT2	16
pT3	20
pT4	8
Grade	
Low grade	3
High grade	41
NDN	
NDN 0	18
NDN 1	10
NDN 2	12
NDN ≥ 3	4
TIT	
FR/FO	34
NE/ST/DI	10

* tissue invasion type was evaluated only in invasive (pT1) tumors

SD – standard deviation

Treg frequency was evaluated in every patient included into this study before tumor removal (Treg-pre), 1 day after the surgical treatment (cystectomy or TUR; Treg-post early), and 7–10 days after the surgical treatment (only for patients treated with cystectomy; Treg-post late).

Type of tissue invasion, which reflects local tumor spread (TIT), and nonclassic differentiation number, which reflects the tendency toward divergent histological differentiation (NDN), were evaluated as described previously ([6] and [5], respectively). Tumor size was determined by multiplying its three macroscopic dimensions (the width and length of the plane of the bladder surface and the depth of infiltration measured at the cross section of the bladder wall). The tumor mass volume obtained for each tumor was classified into one of three categories: (1) small tumors: ≤ 25 cm^3^, (2) medium-size tumors: > 25 and ≤ 99 cm^3^, and (3) large tumors: > 99 cm^3^.

The sections were viewed under Nikon Eclipse 80i light microscope and microphotographs we prepared using Nikon Digital Sight DS Fi1-U2 digital camera and NIS-Elements BR 3.0 software (Nikon Instruments Europe B.V., Badhoevedorp, The Netherlands).

### 4.2 Immunolabeling of Tregs in peripheral blood and flow cytometry

The samples used for the cytometric analysis of Treg populations in the whole blood of patients were prepared using the FoxP3 Staining Kit (Becton Dickinson, Franklin Lakes, NY, USA), according to the manufacturer's instructions and analyzed using a BD FACS Canto II flow cytometer and the BD FACS Diva Software (Becton Dickinson, Franklin Lakes, NY, USA). For each sample, 3 × 10^4^ lymphocytes were collected and gated on an SSC × CD45 dot plot. Subsequently, the populations of CD4 FITC, CD25 APC, and double-positive CD4+/CD25+ cells were distinguished among the lymphocytes, and the gate of FoxP3+ cells was established on the CD4+/CD25^high^+ subpopulation, which are the only cells which exhibit regulatory function in humans [42]. Recently published data identified new population of Treg cells, expressing C-C chemokine receptor 4 (CCR4) and considered CCR+ Tregs as effector Tregs cells [58]. Our preliminary, unpublished results showed about 98% convergence of Treg frequency in CD4+/CD25^high^+ subpopulation and CCR4+ Tregs.

The frequency of Tregs was defined as the percentage of cells with the CD25^high^+/FoxP3+ phenotype in the subpopulation of CD4+ T lymphocytes. In addition to the specific-staining cell analysis, a negative control was used for each sample to determine the frequency of autofluorescence, and an isotypic control, performed for each blood sample, was used to exclude nonspecific staining of the antibodies.

### 4.3 Immunohistochemistry

#### 4.3.1 RCAS1 immunohistochemistry and assessment

For immunohistochemical analysis, 4-μm-thick sections were cut from archival paraffin-embedded blocks and stained with an anti-RCAS1 antibody (Medical & Biological Laboratories Co., Ltd, Naka-ku Nagoya, Japan), as described previously [6, 59]. The RCAS1 immunostaining was evaluated both in tumor cells and in cells of tumor nearest environment: CAFs and TAMs, found within the 2 mm (4 HPF) from the tumor border. The staining intensity of immunolabeled sections was evaluated with reference to the immunostaining of the control, which was scored as strong. Staining intensity was scored from 0 to 3 arbitrary units: negative (0), low (1), moderate (2), or high (3). To increase the objectivity of the data, an expression intensity of 1, as an indicator of low-intensity RCAS1 expression (most subjective), was rejected and was not included in the statistical analysis. The central and border parts of tumors (CPs and BPs, respectively) were defined as described previously [6, 47, 59]. Briefly, central parts were characterized by a low mitotic index and dynamism of growth, whereas border parts were characterized by a high mitotic index, the presence of small nests of tumor cells, inflammatory infiltration, and/or type of stromal modeling pattern.

#### 4.3.2 TILs immunohistochemistry and assessment

In 39 patients with pTa-pT4 urinary bladder cancer, in which Tregs were evaluated in peripheral blood, CD4+ and CD8+ TILs were identified in tumor sections using immunohistochemistry in 4-μm thick sections cut from archival paraffin-embedded blocks. Qualification of patients into this analysis, was followed by verification of formalin-fixed paraffin-embedded tissue blocks quality and the availability of tissue tumor in the section after routine diagnostic procedures. All reagents and equipment were purchased from, Dako (Carpinteria, CA, USA), unless otherwise specified. Antigen retrieval was performed using PT-Link equipment in high pH buffer. Other steps of immunolabelling were done in Autostainer Link 48, as follows: after peroxidase blocking with hydrogen peroxidase, ready-to-use anti-CD4 or anti-CD8 antibodies were applied for 20 minutes. Antigen-linked antibodies were visualized using the the system EnVision+ System-HRP and diaminobenzidine (Dako, Carpinteria, CA, USA). Then cell nuclei were stained with hematoxylin and preparations were sealed in solid medium (Consul Mount; Thermo Fisher Scientific Inc. Waltham, MA, USA). The number of CD4-positive and CD8-positive cells per high-power field (HPF, 400x) was assessed.

### 4.3 Statistical analysis

The differences in Treg frequency in relation to the variables analyzed were assessed using the Mann–Whitney *U* test or the Kruskal–Wallis test. To evaluate statistically significant differences among pairs of data pointed by Kruskall-Wallis test, the Dunn's test was used. For matched data, the Wilcoxon matched-pair test was used. Survival time was assessed using Kaplan–Meier curves. The statistical analyses were carried out using the STATISTICA data analysis software (version 8.0; StatSoft Inc., Tulsa, OK). A *P* value < 0.05 was considered indicative of statistical significance.
